# Emotional violence and maternal mental health: a qualitative study among women in northern Vietnam

**DOI:** 10.1186/s12905-018-0553-9

**Published:** 2018-04-24

**Authors:** Trần Thơ Nhị, Nguyễn Thị Thúy Hạnh, Tine M. Gammeltoft

**Affiliations:** 1 Department of Population, Institute for Preventive Medicine and Public Health, Hanoi Medical University, No.1 Ton That Tung, Khuong Thuong, Dong Da, Hanoi, Vietnam; 2 Department of Anthropology, University of Copenhagen, Øster Farimagsgade 5, DK-1353 Copenhagen K, Denmark

**Keywords:** Depression, Distress, Emotional violence, Ethnographic interviews, Intimate partner violence, Mental health, Pregnancy, Postpartum, Vietnam

## Abstract

**Background:**

Worldwide, intimate partner violence (IPV) during pregnancy is a pressing and prevalent public health problem. Existing research has found close associations between IPV and perinatal mental health, yet little is known about women’s own perceptions of these associations. This study aimed to explore Vietnamese women’s experiences of emotional partner violence and their perceptions of the implications of such violence for their mental health.

**Methods:**

The data were collected through in-depth interviews with 20 women living in Hanoi, Vietnam who had reported exposure to emotional partner violence and attained high depression scores in a prospective cohort study. Ten women were pregnant and ten had recently given birth. The data were analysed by qualitative content analysis.

**Results:**

The women described emotional partner violence as a major life stressor. Their accounts pointed to three particularly significant dimensions of emotional violence: being ignored by the husband; being denied support; and being exposed to controlling behaviours. These experiences affected the women’s sense of wellbeing profoundly, causing sadness and distress. The women’s accounts indicated that experiences of emotional violence were significantly shaped by dominant kinship arrangements: practices of patrilocal residence and principles of patrilineal descent tended to aggravate women’s vulnerabilities to partner violence.

**Conclusions:**

This qualitative study from Vietnam documents close associations between emotional partner violence and perinatal distress, while also pointing to kinship arrangements as particularly significant structural contexts shaping women’s experiences of partner violence. The study findings suggest that effective policies and programs to decrease women’s vulnerability to intimate partner violence must take into account the kinship arrangements that prevail in a given society.

## Background

Worldwide, intimate partner violence (IPV) during pregnancy is a pressing and prevalent public health problem [1–4]. The most widespread form of IPV is emotional violence, defined in operational terms by the World Health Organization as “being insulted or made to feel bad about oneself; being humiliated in front of others; being intimidated or scared on purpose; being threatened directly or through a threat to someone one cares about” [5]. Existing research has found close associations between emotional violence and women’s mental health during pregnancy and in the postpartum period: there is strong evidence that exposure to such violence significantly increases women’s risks of depression and other mental health problems, with far-ranging consequences for the health of themselves and their children [6–9]. The world over, emotional violence and other forms of intimate partner violence pose serious threats to women’s health, dignity and autonomy, while also affecting children, families and communities [3, 5].

In Vietnam, research has documented frequent occurrence of emotional violence: a nationwide study found that the lifetime prevalence of emotional partner violence was 54% and the past year prevalence 25% [10]. Further, several studies have found increased risk of common mental disorders in the perinatal period among women who are exposed to emotional partner violence [11–14]. While the statistical associations between violence and perinatal mental health are well documented, little is known about Vietnamese women’s own perceptions of emotional partner violence and the impact of such violence on their mental well-being. This study therefore set out to explore in qualitative terms how women living in Northern Vietnam experience being exposed to emotional abuse by their husbands and how they assess the impact of such tense intimate relations on their mental health. Since, as the WHO points out, the meanings and implications of emotional violence vary across cultures [5], more in-depth knowledge of the meanings and implications of this form of violence in the context of particular life conditions is needed. In order to facilitate targeted and culturally relevant interventions addressing violence perpetrated within intimate relationships, qualitative insights into women’s own experiences of their everyday lives seem crucial.

## Methods

### Design and recruitment

This study is part of the larger interdisciplinary research project PAVE (The Impact of Violence on Reproductive Health in Tanzania and Vietnam) that explores the impact of partner violence on women’s reproductive health. The overall PAVE project study design included a quantitative and a qualitative component. In Vietnam, the quantitative component consisted of a prospective cohort study involving a total of 1337 pregnant women living in Hanoi’s Dong Anh District. Each woman was assigned a case manager and interviewed four times: at enrolment (at a gestational age of less than 24 weeks); at a gestational age of 30–34 weeks; 24–48 h after delivery; and 4–12 weeks after delivery. For measurement of intimate partner violence, the cohort study relied on criteria established by the World Health Organization [5]. The questions concerning emotional violence explored whether the women had experienced the following behaviours from their husband’s side: insults, belittling/humiliation, intimidation, and threats. The Edinburgh Postnatal Depression Scale (EPDS) was used to assess the women’s risk of perinatal depression. The EPDS includes a 10 item questionnaire with four response categories scored from 0 to 3, yielding a range of 0–30, whereby the greatest values represent pronounced signs of depression [15]. In the present project, women with an EPDS score of 10 or higher were defined as presenting with signs of depression [16]. The qualitative component of the research consisted of in-depth interviews with 20 women who were purposefully selected among the women involved in the cohort study. Criteria for enrolment in the qualitative research were exposure to emotional violence during pregnancy and an EPDS score, either during pregnancy or after birth, at 10 or above. No women declined the invitation to take part in the qualitative study.

### Participants

The 20 women ranged in age from 18 to 37 years (mean age: 26 years). Seven women were pregnant with their first child; nine women were pregnant with their second child, and four with their third child. Seven women had finished secondary school, while 13 had finished tertiary school and above. Two women reported unemployment status, while the remaining worked mainly in factories or as farmers or traders. When getting married, all women had left their natal family and moved into the household of their husband and his parents. Later, two couples had changed their residence to live with the wife’s parents instead, as they lived in an area where employment opportunities were better, while four couples had set up a separate household and now lived in a two-generation household, but still in close vicinity to the husband’s parents. At the time of the interview, then, 14 of the women shared a household with their husband and his parents, while two lived in a three-generation family with their own parents, and four lived in a nuclear family. Eighteen of the women were interviewed once, and two women were interviewed twice (Table 1).

**Table 1:** Descriptive characteristics of study participants (*N* = 20)

Characteristics	No. of women	% of total
Age (years)		
Mean (SD)	26 ± 4.6	
Min	17	
Max	37	
Place of birth		
Present community in Dong Anh District	8	40.0
Another community in Dong Anh District	8	40.0
Another district/Another province or city	4	20.0
Occupation of women		
Government employee Private company /Organization employee	1	5.0
Work in private company	5	25.0
Farmer	5	25.0
Small trade	4	20.0
Student	3	15.0
Unemployed	2	10.0
Level of education		
Secondary school (grade 6–9 years)	7	35.0
High school (grade 10–12 years)	7	35.0
University/college ((> 12 years)	6	30.0
Living arrangement in relation to partner		
Married and living together	20	100.0
Living arrangement in relation to family of birth*and in-laws (*n* = 1274)		
Living in nuclear family	4	20.0
Living with family of birth	2	10.0
Living with family in-law	14	70.0
Number of children		
1	7	35.0
2	9	45.0
3	4	20.0

### Data collection and handling

The data were collected through in-depth interviews using an open-ended, semi-structured interview guide. The interviews were conducted in the period from August 2014 to August 2015 by the first and the third author; one interview was conducted by all three authors. The interviews were conducted in the women’s own homes, with only the researchers and the woman present. Each interview started from a presentation of the research and its purposes, followed by informal conversation about family, work, or other topics. The women were then encouraged to tell their “love stories”; that is, the story about how they had met their husband and how the relationship had developed. These stories often included narratives about partner violence and about the stresses and joys of childbearing. During each interview, we went over the woman’s cohort study questionnaire, encouraging her to tell us in more detail about the instances of partner violence and the mental health states that she had reported during the quantitative interviews. These conversations offered more detailed and contextualized insights into the women’s feelings and life situations than the quantitative questionnaires had been able to provide. Each interview lasted between 90 and 120 min and was voice recorded and transcribed in Vietnamese.

### Data analysis

After each day of field research, the researchers took detailed field notes, including both verbal information gained and other observations. The data were synthesized and interpreted by applying a content analysis strategy [17]: all field notes and transcriptions were read and coded for instances and types of emotional violence; for women’s descriptions of their own mental health states; and for the connections between violence and mental health. The codes were grouped into summary concepts with a concise name. The analysis was performed on the Vietnamese language transcripts and only the interview quotes selected for this article were translated into English.

### Ethical considerations

Ethical approval of the research was obtained from Hanoi Medical University Research Ethics Committee (Decision 137/HMU IRB dated November 29, 2013). The WHO ethical and safety recommendations for researching domestic violence against women were also followed [18]. The women were informed in detail about the purpose of the study. When they agreed to participate in the quantitative part of the research, they signed an informed consent form, and for the qualitative research, verbal consent was given. Women experiencing intimate partner violence were given a list of local organizations that could help them to avoid partner violence and provide them with counselling services. Mental health care services are highly limited in Vietnam [19], but possibilities for psychological counselling (mainly in tertiary level hospitals and in the private health care sector) were brought to the women’s attention. To protect the anonymity of the women, all personal names used in this article are pseudonyms.

## Results

### Experiences of emotional violence

All women in the study described the emotional violence that they lived with as a huge life problem. The stories that they told us were usually stories of an initial love affair and a short period of marital bliss, followed by a sudden change in their husband’s behaviour towards them. From being warm and attentive during the time of courtship, he suddenly became cold, indifferent, and distant, sometimes talking to them in derogatory ways. To illustrate how the women experienced such behaviours, we begin by presenting the case of Lan. We invited Lan to take part in the qualitative research because the cohort study interview with her revealed an EPDS score of 16 as well as extensive experience with emotional violence. When we called Lan to make the appointment for an interview, she immediately agreed to take part. On August 25, 2014, we went to visit her in the Dong Anh town where she lived.

#### Lan’s story

When we met her, Lan was 36 years old and 38 weeks pregnant with her third child, a girl. She shared a house with her husband, their two children, and her parents-in-law. Lan’s husband worked as an iron welder, and she herself ran a small café in the front room of their house, selling home-made sticky rice and tea. Since most of her customers came in the early morning, Lan had to get up very early every day, preparing for work. When we reached Lan’s house, it was 10 am and her café was quiet. Offering us each a cup of green tea, Lan seated us in the rattan furniture in the back of the room. She told us that she had felt much more tired during this pregnancy than during her first two pregnancies. Unlike her first pregnancies, this one was not planned. Also, with two small children and two elderly parents-in-law to take care of, alongside her work running the café, Lan had to work very hard during this pregnancy. She had felt quite weak, had vomited a lot, had difficulties sleeping, and suffered from hypertension.

We asked Lan to tell us about her marital life, beginning from the time when she and her husband met each other. At that time, Lan said, her husband had been so sweet and kind to her. He lived a simple life. Unlike many other men, he did not have a motorbike; instead, he biked the nine kilometres to her parents’ house to visit her. She fell in love with him, and they got married. Since the day of their marriage, Lan feels, her husband has become colder towards her day by day. She does not understand why, but he seems to act completely differently with other people than he does with her. With his parents, he is always gentle and respectful, treating them with love and care. If they get ill, he asks constantly how they are, expressing sincere concern about them and buying medicine for them at the local drugstore. He is also very friendly with his neighbours, who see him as a kind man. When he is with Lan, however, he seems to be a completely different person. When at home, he ignores her; he does not talk to her and acts as if she is not present; a non-person of no value. He leaves all housework to her, failing to help out with any domestic chores. When she is ill, he does not ask how she is and does not do anything to support her. Lan feels that there is no love in their marriage anymore. Her husband’s coldness towards her is particularly painful to her because she is pregnant:

When I asked him to drive me to the hospital for an antenatal check-up, he refused. He does not seem to care about the little one or me at all. It’s so strange, because other people see him as a kind man, but at home, we never talk to each other. Sometimes I look at other couples. They talk to each other, like friends. But my husband and I never talk. When the children are ill, it’s only me who takes care of them. If they wake up at night, my husband just continues sleeping. The children cry, but he just sleeps. This makes me feel very angry. Sometimes, if I have problems with my health, I tell him that I’m not feeling well. But he does not care.

Sharing a household with his parents also places strain on the relationship between Lan and her husband. At first, when she moved into this house after their wedding, Lan found it very difficult to live with his parents: she found them old-fashioned, and she felt that they were constantly observing her and correcting her. They commented on behaviours that seemed of minor importance to Lan, such as the way she washed the dishes and the amount of water that she used. If she returned from an errand and failed to greet them in the way that her husband found correct, he would scold her in a harsh tone.

When moving to live here, Lan constantly missed her parents’ house where she had felt much more relaxed and at ease – the atmosphere in her husband’s home was so different and much more tense. Even though she had lived here for several years now, there was no one in the family or in the local area that she felt she could really trust, as everyone here was closely related to her husband and his family: “To tell you the truth,” she said, “I don’t confide in anyone. I’ve married a man who lives far from my own home, so I have no one to confide in. I also don’t hang out with anyone here. I just stay at home and work.” Even though he himself does not want to talk to her, Lan says, her husband gets angry if she talks too much to male customers or other men: “One day when my phone rang, my husband grabbed it. The person on the other end did not respond. Then my husband asked me, ‘Why is the person not speaking?’ After that my husband became very angry. He shouted, in a really coarse tone, and said that he would trace the phone number to find out who had called.”

Living in this marriage, Lan feels deeply unhappy. She cries a lot and thinks a lot. “Sadness is always there,” she says, “it’s always haunting me.” She has often thought of ending the marriage, but she stays for the sake of the children, feeling that a divorce would make their lives very difficult. She also feels that if she leaves this house, there is nowhere else that she can go. Even though her mother has invited her to return to her parents’ house, this house now belongs to Lan’s brother and his wife, and Lan does not feel she would be welcome there. So she endures: “Whatever one’s life conditions are like,” she says, “one must endure those conditions.”

Lan’s story resembles many other stories that we heard when talking to women who lived with emotional partner violence. From our analysis of the women’s narratives of their marital lives, three forms of emotional abuse emerged as the most common and painful to women: being ignored by the husband; not being supported by the husband, particularly in case of family conflicts; and being the victim of controlling behaviours. In Lan’s case, all three problems seemed to coincide, whereas in other cases, one of these problems was the most pressing. We shall now go on to a presentation of each of these behaviours as the women described them.

#### Being ignored

Like Lan, many of the women struggled with feelings of being ignored by their husband. They told us that their husband seemed to have no interest in talking to them; even though he might be lively and talkative in the company of others, he would turn silent as soon as they were alone, failing to respond to the things that his wife said to him. To many women, this behaviour from their husband’s side seemed to be aggravated by two conditions: firstly, by the fact that they were pregnant or had recently given birth, and secondly, by the fact that – in accordance with local kinship prescriptions – they had moved to live in their husband’s household after getting married. Being pregnant, or having recently given birth, seemed to increase the women’s expectations regarding marital intimacy. Since the days of courtship, they had felt a need to be in touch with their husband, but now they needed it more than ever. The women generally experienced their pregnancies as a time of vulnerability and transformation, a time where they needed to be in contact with the father of the child that they expected. Liên, who was 33 years old when we met her, described her marriage in these words:

These days we barely talk to one another or look at each other. It seems as if he hates me. He does not care about me at all. But I’m pregnant, so I need some encouraging words so much. Everyone encourages me to be strong and take care of my health, but frankly, I feel so unhappy when I receive no words from my husband. Even if I speak very gently to him, he just shouts back at me.

Further, living in their husband’s household often seemed to increase the women’s vulnerability to their husband’s behaviours. Like Lan, many women had to leave their natal home behind when getting married – the people who used to be their supporters and best friends now lived in another locality, and they found themselves in a household and a village populated by strangers. In case of marital conflicts, other household and community members would usually side with those they knew best: the husband. In many cases, this residential situation created deep feelings of social isolation – feelings that were aggravated in situations where the woman, as in Lan’s case, did not have employment outside the house.

#### Being denied support

Like Lan, many women in the study felt burdened by their husband’s lack of support for them. The women’s narratives indicated that the support they lacked could be either of a practical, an emotional, or a social nature. Some women expressed needs for more practical support, while others longed for more emotional or social support. The needs for practical support might concern, for instance, transport to antenatal care or other health check-ups, help with domestic chores, assistance with child care, or other forms of sharing of tasks that were usually considered female obligations. Many women also missed receiving more emotional support from their husband. They felt that their husband did not want to listen to them or take their concerns seriously, and that he seemed uninterested in helping or consoling them if they felt sad or distressed. Finally, many women expressed needs for more sustained social support from the husband. This need for social support was particularly evident in cases of family conflicts. Living together with, or nearby, their mother-in-law threw many women into domestic disagreements and conflicts: like Lan, many felt that their mother-in-law lived in old-fashioned ways, and that her recommendations regarding childcare and housekeeping were outdated, belonging to an earlier era. At the same time, the older woman would usually expect to be in authority: this was her household, and the daughter-in-law was a very new member of it. Many women therefore told us of clashes and conflicts between themselves and their mother-in-law; conflicts that seemed to be particularly painful when they occurred in the vulnerable times of pregnancy and the postpartum period. The disagreements often centered on childcare questions, such as which supplemental food to give the child or how the child should sleep. In such disagreements, the women hoped that their husband would support them – but in many cases, he opted to side with his mother. One 25-year old woman named Hạnh told us of a conflict between her and her mother-in-law that resulted in her husband’s humiliation of her:

One day when I was nursing my baby, my mother-in-law was shouting a lot at me and I had a really bad headache. She went out and came back with some medicine and asked me to take it. I agreed, but I also said that her shouting made me feel worse. Then she got really angry, scolding me in a loud voice and telling me how rude I was. Then she told me to leave the house and let the baby stay. When I told my husband the story, he scolded me in a rude language, saying things such as ‘Go and fuck your mother,’ and shouted, ‘Shut up, if you don’t want me to beat you right now.’

Again, the combined conditions of being pregnant/having recently given birth and living in the husband’s household together with his parents seemed to aggravate the women’s vulnerability to emotional partner violence.

#### Being controlled

The last dimension of emotional violence that emerged particularly frequently in the women’s accounts concerned feelings of being placed under control. Many women told us that their husband was very jealous, always keeping a close eye on them, interrogating them about their whereabouts, and sometimes checking their mobile phones to see whom they had talked to. Twenty-five year old Lý said:

My husband doesn’t allow me to have much contact with others. He does not want me to go out, even if he is present himself he does not like it. He says, ‘Now you are a married woman, so I think you should refrain from hanging out with your friends. You see, it’s so meaningless...’ He threw out my phone immediately when I was Facebook surfing... He is jealous. Now, he no longer allows me to use a cell phone.

Another woman, Duyên, who was 18 years old and newly married, told us about how isolated she felt living in her husband’s household. She missed her natal parents and siblings intensely and sometimes regretted that she had married her husband. He had been very sweet to her before they got married, but now his attitude toward her had changed completely. Since she had given birth to her child, she felt that she was losing all self-confidence and trust in herself due to the criticism that her husband directed at her and due to her lack of social network in the area where she was now residing:

My husband criticizes me for being fat and tells me to lose weight. I feel so sad, and I just want to stay at home with my child. Due to my appearance, I don’t want to go out and get attention from others. Before we got married, my husband never spoke like this, but these days, he criticizes me because I’ve gained weight after the birth. He says that I eat too much and that my skin is too dark.

Seeking to maintain domestic stability and peace, most of the women who experienced controlling behaviours from their husband’s side tried to adapt to his wishes by not going out too much and by not challenging the limitations that he placed on them. Combined with the fact that they were either pregnant or had recently given birth, this often placed the women in a situation of existential isolation. Even though most women had daily contact with others in their household concerning practical matters such as shopping or cooking, they often longed for the trustful and intimate social relations that they had to friends and natal relatives prior to getting married.

### Consequences of emotional violence for women’s mental well-being

All women in this study felt that their husband’s negative behaviours towards them – his ignoring them, failing to support them, or controlling them – drained them of energy both physically and emotionally, seriously affecting their overall health. When getting married, the women said, they had never imagined that married life would turn out like this. Most of the women were constantly thinking about their plight, about why their husband acted like this, and about what they could do to improve the situation. This constant thinking, they felt, affected their health deeply, burdening their bodies and minds. Twenty-six-year-old Thanh put it like this:

I suffer from headaches all the time. I think too much, so the blood cannot come to the brain. I think about my husband and thinking about him makes me feel so depressed. Then I do not want to eat anymore. Sometimes I just eat twice in two or three days, sometimes I don’t even have breakfast… I cannot sleep, and I’m easily frightened. This thinking is always on my mind. It stresses me.

Twenty-five year old Hạnh told us how, as a result of her husband’s way of treating her, she had become increasingly unhappy and depressed. She said:

I keep thinking all the time and I always feel that something bad will happen to me. Thinking too much gives me nightmares. I feel that people avoid talking to me, and I rarely go out. I just stay at home and sleep all day. I feel depressed and isolated. Now I’m totally different from the person I used to be. Perhaps I’m at the bottom of my life right now.

Many other women reported feelings of sadness similar to those that Hạnh described. Also 18-year-old Duyên said that she used to be a completely different person from the one that she had now become. During her pregnancy, she said, she had cried a lot and experienced overwhelming sadness, as she thought about how well her husband had cared for her before. Now, he seemed to completely disregard her, spending all his time hanging out with his friends, hardly even touching her:

Before, I never used to cry. But I started to cry when I got pregnant. I cried about my husband’s anger or his scolding me. Perhaps I suffer from depression. I think depression is the feeling of not having anyone to confide in, anyone to share with… I read some articles about pregnant women who suffered from depression because they could not share family controversies with anyone. According to these articles, pregnant women are more prone than others to depression.

Like Duyên, many women reported feelings of sadness and depression and a loss of interest in food and other things that they had used to enjoy. Twenty-eight-year-old Tâm, for instance, said:

I have no appetite, so I eat less. I feel constantly sad, and there is nothing that I find interesting anymore. All I want to do is to take care of my children. I’m not interested in household tasks anymore either. Before I would cook delicious meals and teach my children how to cook them, but when my husband and I are not on good terms, I don’t want to do anything anymore. This directly affects my children.

While low moods – manifesting in too many thoughts, feelings of sadness and depression, and loss of interest in things that they used to enjoy – were described by all women in the study, four women also reported considering suicide and other forms of self-harm. One woman mentioned that she had thought of cutting an artery in her wrist and another took sleeping pills in search of a mental escape. The women who considered suicide all ended up not attempting this out of concern for what would happen to their children if they were not there to care for them anymore. Liên explained:

One time, I thought that I would use a knife to cut my hand. My husband knew about that, but he didn’t care. He even said to me ‘You should go ahead and do that.’ That made me so angry. And then I thought again: ‘If I die my children will not be cared for by anyone.’ So, I ended up not cutting myself because I love my children. My children need me.

## Discussion

This study set out to investigate Vietnamese women’s own perceptions of emotional partner violence during pregnancy and in the postpartum period and the effects of such violence on their mental health. The results showed that in women’s own experience, exposure to partner violence affected them profoundly, generating deep feelings of sadness and tension and a tendency to “think too much”. These findings confirm what quantitative studies of the associations between intimate partner violence and perinatal health among women in Vietnam have shown [11–14]. Through the use of qualitative methods, however, the present study has provided more nuanced insights into what emotional violence means in practice as it is lived and experienced by northern Vietnamese women in the context of their day-to-day lives.

While emotional violence, in WHO’s operational definition of the term, can refer to a variety of different acts – ranging from humiliation to intimidation to threats of harm – the acts that the women in this study emphasized the most when describing their husband’s negative behaviours towards them fell into three distinct categories: the women felt ignored, felt they were denied support, and felt controlled. These three manifestations of emotional violence are closely interrelated: they all reflect a marital situation where the woman feels that her personal needs are being set aside – where she is treated as a non-person, someone unworthy of attention, support, and respect. Existing studies of domestic violence in Vietnam have documented similar patterns of strained marital situations in which husbands exercise and maintain male authority by acting violently against their wives and/or ignoring their needs [20–23]. This research has most often interpreted such abusive marital situations as matters of interpersonal relations between men who exercise violence and women who are subjected to it. This has led to important investigations of dominant gender ideologies in Vietnam – ideologies that emphasize women’s obligations to maintain marital harmony and men’s positions as “pillars” of the family. These notions of masculinity and femininity draw on long-standing Confucian moral prescriptions for appropriate gender behaviours and are re-interpreted and re-circulated in new guises by present-day social authorities [24, 25].

While interpersonal male-female relations and dominant gender norms are clearly important elements in an understanding of the prevalence of emotional partner violence within Vietnamese households and the effects of such violence on women’s mental health, our research indicates that structural factors of another nature must be taken into account too. The women’s accounts indicated that kinship arrangements set particularly important structural conditions for the day-to-day exercise and experience of emotional partner violence. Gender relations in northern Vietnam are, our findings show, embedded in larger arrangements of male-oriented kinship [26, 27]. This is in line with Montesanti’s observation that “gender is inescapably embedded in social systems and institutions” [26].

In northern Vietnam, prescribed kinship is patrilineal and patrilocal [27, 28]. In practical terms, this means that children are considered to belong to their father’s lineage (descent is reckoned patrilineally) and that women will leave their natal families and move to live with or nearby their husband’s family when getting married (marriages are patrilocal). All women in this study had moved into the household of their husband and his parents when getting married. Fourteen women still lived in this household, while four lived in nuclear families in close vicinity to their husband’s parents and other relatives. Our findings show that these living conditions – having to leave their own natal family behind and move into a household of strangers – set very particular conditions for the women’s marriages and their experiences of marital strain. First, the women’s vulnerability to emotional partner violence seemed to deepen due to the social isolation that many of them experienced. The fact that their natal relatives and childhood friends were now living somewhere else tended to increase the women’s dependency on their husband: he was their link to this new family; the one who had brought them here. If he ignored them or failed to support them, they risked finding themselves in a position of deep isolation. This risk was aggravated by the fact that the women were pregnant or had recently given birth, and therefore more closely tied to the domestic arena than they might otherwise have been. Second, the women’s possibilities of confiding in others and achieving help and support were constricted by the fact that the man who was treating them badly was the son or brother of other household members, and a long-term community member. In this situation, expressing their grievance or seeking support could easily be interpreted as an expression of a lack of loyalty, and the chances that other household members would side with the woman were not large. Thirdly, in case of conflicts between the woman and her mother-in-law – which are ubiquitous in many parts of Asia [29] – the man will often feel culturally obliged to support his mother, a tendency that may place further strain on the marital relationship. Fourthly, as descent is reckoned patrilineally in northern Vietnam, inheritance usually follows male lines and daughters tend to inherit only a minor share of their parents’ assets [30, 31]. This makes it more difficult for women to protest against violence or to leave an abusive marriage, as the land, the house and other property will usually belong to their husband. All this indicates, in sum, that the very deep emotional frustration expressed by women in our study – sometimes even including suicidal ideation – must be seen in the context of the male-oriented kinship arrangements which deepened women’s vulnerability to emotional violence and other abusive behaviours from their husband’s side.

Even though previous studies, conducted both in Vietnam and elsewhere, have found that domestic tensions and household conflicts increase women’s risks of perinatal mental health problems [32, 33], few studies have pointed to the systematic arrangements of kinship - in the form of patrilineality and patrilocality – that underlie abusive marital relations and shape the impact of such strained relations on women’s lives. Our findings indicate that the time has come to pay closer attention – in research, policy, and health care programming – to the impact of kinship patterns on women’s everyday lives, including on their experiences of partner violence and their capacities to handle such violence.

## Limitations of the study

This study has several limitations. Firstly, both violence and depression are sensitive topics that are likely to involve complex personal feelings such as guilt, embarrassment and shame. It is therefore possible that the women held back some aspects of their experiences. Secondly, this sample was drawn from one particular group of women living in Dong Anh District in Hanoi, Vietnam and the empirical findings may therefore not be generalizable to mothers from other areas in Vietnam.

## Conclusions

Relying on qualitative methodologies, this study has investigated how women in northern Vietnam experience emotional partner violence and explored the impact of such violence on women’s perceived mental health. The results show that as experienced by the women, key dimensions of emotional partner violence included being ignored; being denied support; and being exposed to controlling behaviours. Living in daily exposure to such behaviours affected the women’s perceived mental health profoundly, causing sadness and distress. The research found that dominant kinship arrangements played crucial roles shaping women’s experiences of emotional violence and its effects on their wellbeing; in particular, practices of patrilocality and patrilineality tended to increase women’s vulnerability to emotional violence and to limit their capacities to act against it. These findings point to the necessity of extending the predominant focus on gender in research on intimate partner violence, addressing also the larger systems of kinship in which gender norms and -relations are embedded. Further, health care policies and programmes addressing intimate partner violence might benefit from increased attention to systems of kinship, for instance through the implementation of interventions that involve the individuals and social networks that provide married women with informal support, such as their natal kin [34], their neighbours [35], or their childhood friends.

## Abbreviations

DANIDA: Danish international development agency
EPDS: Edinburgh postnatal depression scale
IPV: Intimate partner violence
IRB: Institutional review board
PAVE: The impact of violence on reproductive health in Tanzania and Vietnam
WHO: World Health Organization
